# Evaluation of Screening Tests in Bavarian Healthcare Facilities during the Second Wave of the SARS-CoV-2 Pandemic

**DOI:** 10.3390/ijerph18147371

**Published:** 2021-07-09

**Authors:** Christina Tischer, Carolin Stupp, Patrick Janson, Kristina Willeke, Chu-Wei Hung, Jessica Flöter, Anna Kirchner, Katharina Zink, Lisa Eder, Christina Hackl, Ursula Mühle, Manfred Weidmann, Uta Nennstiel, Joseph Kuhn, Christian Weidner, Bernhard Liebl, Manfred Wildner, Thomas Keil

**Affiliations:** 1 State Institute of Health, Bavarian Health and Food Safety Authority, Eggenreuther Weg 43, 91058 Erlangen, Germany; Carolin.Stupp@lgl.bayern.de (C.S.); patrick.janson@lgl.bayern.de (P.J.); kristina.willeke@lgl.bayern.de (K.W.); Chu-Wei.Hung@lgl.bayern.de (C.-W.H.); jessica.floeter@lgl.bayern.de (J.F.); anna.kirchner@lgl.bayern.de (A.K.); katharina.zink@lgl.bayern.de (K.Z.); lisa.eder@lgl.bayern.de (L.E.); cthackl@web.de (C.H.); Ursula.Muehle@lgl.bayern.de (U.M.); uta.nennstiel@lgl.bayern.de (U.N.); Joseph.Kuhn@lgl.bayern.de (J.K.); bernhard.liebl@lgl.bayern.de (B.L.); manfred.wildner@lgl.bayern.de (M.W.); thomas.keil@lgl.bayern.de (T.K.); 2 Institute of Clinical Epidemiology and Biometry, University of Wuerzburg, Josef-Schneider-Str. 2, 97080 Wuerzburg, Germany; 3 Pettenkofer School of Public Health, Ludwig Maximilians University, Marchionistrasse 15, 81377 Munich, Germany; 4 Institute for Medical Information Processing, Biometry, and Epidemiology—IBE, Ludwig Maximilians University, Marchionistrasse 15, 81377 Munich, Germany; 5 Institute of Microbiology and Virology, Medical School Brandenburg Theodor Fontane, Universitätsplatz 1, Gebäude 14, 01968 Senftenberg, Germany; Manfred.Weidmann@mhb-fontane.de; 6 Midge Medical GmbH, Colditzstarße 34-36, 12099 Berlin, Germany; 7 Bavarian Health and Food Safety Authority, Eggenreuther Weg 43, 91058 Erlangen, Germany; christian.weidner@lgl.bayern.de; 8 Walther Straub Institute of Pharmacology and Toxicology, Faculty of Medicine, Ludwig Maximilians University, Pettenkoferstrasse 12, 80336 Munich, Germany

**Keywords:** SARS-CoV-2, asymptomatic screening, RT-PCR, antigen testing, infection surveillance

## Abstract

Due to the lack of data on asymptomatic SARS-CoV-2-positive persons in healthcare institutions, they represent an inestimable risk. Therefore, the aim of the current study was to evaluate the first 1,000,000 reported screening tests of asymptomatic staff, patients, residents, and visitors in hospitals and long-term care (LTC) facilities in the State of Bavaria over a period of seven months. Data were used from the online database BayCoRei (Bavarian Corona Screening Tests), established in July 2020. Descriptive analyses were performed, describing the temporal pattern of persons that tested positive for SARS-CoV-2 by real-time polymerase chain reaction (RT-PCR) or antigen tests, stratified by facility. Until 15 March 2021, this database had collected 1,038,146 test results of asymptomatic subjects in healthcare facilities (382,240 by RT-PCR, and 655,906 by antigen tests). Of the RT-PCR tests, 2.2% (n = 8380) were positive: 3.0% in LTC facilities, 2.2% in hospitals, and 1.2% in rehabilitation institutions. Of the antigen tests, 0.4% (n = 2327) were positive: 0.5% in LTC facilities, and 0.3% in both hospitals and rehabilitation institutions, respectively. In LTC facilities and hospitals, infection surveillance using RT-PCR tests, or the less expensive but less sensitive, faster antigen tests, could facilitate the long-term management of the healthcare workforce, patients, and residents.

## 1. Introduction

The World Health Organization (WHO) declared the global spread of the coronavirus disease (COVID-19) caused by a novel beta coronavirus (SARS-CoV-2) a public health emergency of international concern in January 2020 [1]. Being one of the 10 deadliest pandemics in history, the ongoing pandemic has significantly impacted morbidity and mortality worldwide [2]. To date, 178,837,204 confirmed cases of SARS-CoV-2, including 3,880,450 deaths, have been reported worldwide, with 3,723,798 confirmed cases in Germany (as of 22 June 2021) [3]. The pandemic is placing unprecedented and continuous strain on medical facilities [4]. Apart from healthcare professionals [5,6], residents of long-term care facilities are at particular risk [7,8,9,10]. A higher proportion of age-related pre-existing illnesses, living in close proximity to one another, and close physical contact to healthcare workers and caregivers are contributing factors.

In Europe, several SARS-CoV-2 test approaches have been established in the first year of the pandemic, such as testing being limited only to symptomatic individuals [11], but also mass population-screening concepts [12,13], all with the primary aim to identify infection events timely and to prevent uncontrolled viral transmission. Intriguingly, results of testing within long-term care facilities revealed a high rate of asymptomatic infections among healthcare professionals and residents, questioning the usefulness of symptom-based testing alone [14,15]. 

In Germany, the state of Bavaria, located in the southeast of the country, was hit first and hardest of all federal states during the first wave of the pandemic [16,17]. As a result, the State of Bavaria established a comprehensive corona testing strategy for their 13 million inhabitants, including unconditional free voluntary screening tests for all asymptomatic residents at a local doctor’s office in June 2020 [18,19]. The costs for these asymptomatic tests are reimbursed by the federal state and the state of Bavaria, whereas testing of symptomatic persons is covered by the individual’s health insurance company. In addition, and next to compulsory testing in case of outbreak situations, the concept also involves voluntary screening tests for asymptomatic employees of all public and private hospitals and rehabilitation institutions as well as LTC facilities in the context of work based on particular criteria, such as high job exposure or increased local incidences [19]. To this, the occasion-related and preventive tests carried out by the mobile teams of the Task Force Care Homes since April 2020 has to be added [20]. 

The current pandemic situation is unrelentingly critical, specifically for healthcare professionals as well as patients and residents within medical institutions and LTC facilities. Therefore, we aimed to evaluate the Bavarian online database “Meldeportal für Bayerische Corona-Reihenuntersuchungen (BayCoRei)”, which was launched in July and August 2020. Its main purpose is to monitor the implementation of the Bavarian testing directive. The primary scope of the present analysis was to examine the total number and the proportion of positive results regarding both reverse transcriptase polymerase chain reaction (RT-PCR) and antigen tests (since November 2020, calendar week 45) of asymptomatic staff, patients, residents, and visitors in hospitals and LTC facilities. Our secondary aims included describing the regional differences, comparing the seven Bavarian districts, regarding their utilization of the free screening tests, and ultimately, evaluating the temporal trends in the number of tests and positive rates, stratified by type of healthcare institution and person group. Furthermore, we compared the official 7-day SARS-CoV-2 incidence per 100,000 inhabitants in Bavaria with the weekly number and percentage of positive tests.

## 2. Materials and Methods

### 2.1. The Bavarian Corona Testing Strategy

The Bavarian test strategy includes (i) preventive testing for visitors, patients, and residents, in institutions such as hospitals, rehabilitation institutions, and LCT facilities; (ii) preventive testing of healthcare staff working in high-risk areas of hospitals (emergency departments, intensive care units, etc.) or if the local community incidence is >50 per 100,000 inhabitants over the last 7 days; and (iii) preventive testing for employees working at critical institutions (e.g., rescue and first-aid organisations) [19]. 

Apart from the voluntary test offers in Bavaria, testing asymptomatic people by RT-PCR has been obligatory for the (re-)admission of (new) residents into LTC facilities [21,22], and by antigen tests for staff of LTC facilities (at least twice per week), since 8 December 2020 [23]. In general, and since October 2020, antigen tests were recommended for staff, inhabitants, patients, and visitors of LTC facilities as well as hospitals according to the national test strategy. RT-PCR tests were mandatory only in case of an outbreak, or in order to confirm a positive antigen test result [24].

### 2.2. The Bavarian Online Database BayCoRei

**Figure 1:**
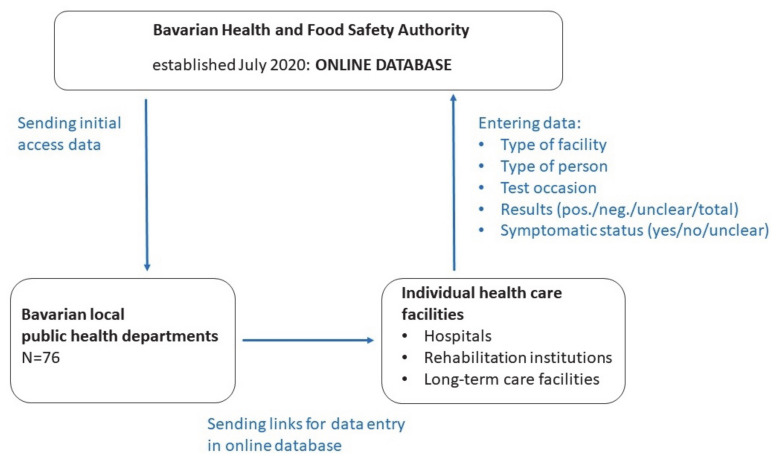
Overview of the interplay between the different actors and the data collection, processing, and sampling procedure.

The online database is a systematic and anonymized test surveillance instrument, with the aim of monitoring regional and temporal patterns of the screening results (Figure 1). Any medical institution and facility that was interested in the free screening tests offered by the State of Bavaria for asymptomatic staff, patients, residents, and visitors had to sign a specific contract with their local public health office before qualifying for the reimbursement of costs for the test kits and procedures.

The healthcare institutions are required to enter the number and results of the tests on a weekly basis in the online database (general practice (GP) offices monthly). Information on RT-PCR and antigen testing for SARS-CoV-2 is collected for several types of medical and public facilities, including GP offices and local test centres. Apart from prevailing outbreak situations, test results are indicated as regular medical screenings for mainly preventive purposes among asymptomatic persons with respect to hospitals and rehabilitation institutions. Tested groups of persons comprise staff, patients (outpatient surgeries in hospitals and before patient admissions in rehabilitation institutions), residents, visitors, and other (e.g., self-employed physiotherapists). Information is updated weekly for the previous calendar week per facility and made available to the Bavarian state ministry of health, all seven district governments, and all 76 local public health offices (Figure 1). It is important to note that the facilities report back individual anonymized data; however, the online database administers data in an aggregated form. Further, multiple testing is possible for the same person.

### 2.3. Study Design of the Current Investigation

The current study aims to give a descriptive overview of the data entered into the Bavarian online database BayCoRei over a period of seven months and thereby encompassing the 2nd wave of the pandemic in 2020 and 2021. Here, we focus on reporting the screening results of testing asymptomatic persons in hospitals, rehabilitation institutions, and LTC facilities. Both the RT-PCR and antigen test results will be depicted together. Given the explorative character, no causal statements or inferences could be derived.

### 2.4. Statistical Methods

Results are either presented summarized per calendar week (for RT-PCR testing from calendar week 36 in 2020 to calendar week 10 in 2021, and for antigen testing from calendar week 45 in 2020 to calendar week 10 in 2021), or accumulated over the total observational period. In general, positive rates of RT-PCR and antigen testing were calculated as positive tests divided by total tests and presented in percentage (%) with the corresponding 95% confidence intervals (95%CI). 

All statistical analyses were performed using the statistical software R, version 4.0.2 [25]. The following information was shown, based on frequency analyses: (1) an overview of the accumulated SARS-CoV-2 screening tests and positive rates among asymptomatic persons in healthcare institutions in Bavaria (Germany); (2) a description of the regional differences comparing the seven Bavarian districts; (3) an evaluation of the temporal trends in the number of tests and positive rates, stratified by the type of healthcare institution and person group; and (4) a comparison of the reported test results with the actual pandemic situation in terms of the 7-day incidence per 100,000 in the State of Bavaria [26]. 

## 3. Results

### 3.1. Reported Test Data in Comparison with Current Statistics 

According to current statistics, the State of Bavaria has 347 hospitals, 250 rehabilitation institutions [27], and 2747 LTC facilities [28,29]. As of 15 March 2021, the online database for the screening tests included data from 157 hospitals, 115 rehabilitation institutions, and 856 LTC facilities, which were instructed with testing according to the Coronavirus-Testverordnung (TestV) [30,31] (data not shown). Regarding serial screening, 67% of the hospitals, 69% of the rehabilitation institutions, and 48% of the LTC facilities have reported back in response to the provided link from the respective local public health department (data not shown).

### 3.2. Overview of Accumulated Screening Tests and Positive Rates

Since the implementation of the online database in July 2020, a total of 1,038,146 RT-PCR and rapid antigen test results have been entered (until 15 March 2021). RT-PCR tests were mostly used in hospitals, whereas antigen tests were predominantly used in LTC facilities (Table 1). Over the 7-month observation period, 8380 RT-PCR tests (2.2% of 382,240) and 2327 antigen tests (0.4% of 655,906) of asymptomatic staff, patients/residents, and visitors were identified as being positive for SARS-CoV-2.

In general, RT-PCR and antigen testing were more prevalent in hospitals than in the other institutions and among asymptomatic staff as opposed to asymptomatic patients/residents. Further, the cumulative RT-PCR positive rate was found to be consistently higher than the positive rate of antigen tests in all person groups in the three types of facilities. The amount of RT-PCR testing in asymptomatic visitors was very low for all types of facilities (not exceeding 0.7%). Antigen testing of asymptomatic visitors was most frequent in LTC facilities (11%) as compared to hospitals (4%) and rehabilitation institutions (3%) (Table 1). Therefore, considering the temporal patterns by weekly results, we are presenting only the antigen test positivity rate for asymptomatic visitors of LTC facilities.

### 3.3. Comparison of Regional Differences in the State of Bavaria

**Figure 2:**
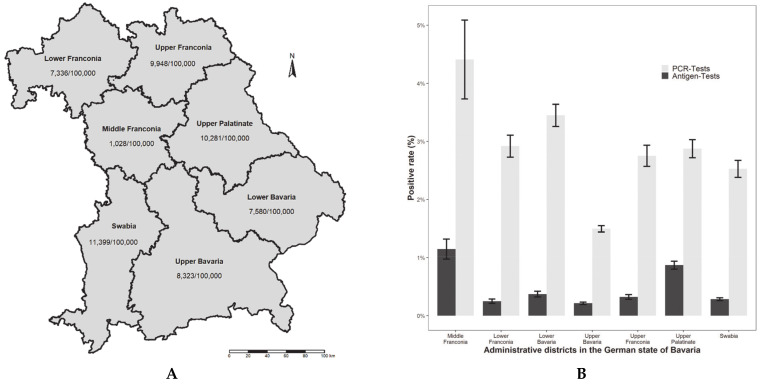
**A**. Accumulated number of serial screening tests (RT-PCR and antigen tests combined) of asymptomatic staff, patients/residents, and visitors in healthcare facilities in relation to 100,000 inhabitants for all seven administrative districts in the German state of Bavaria. **B**. Accumulated positive quotes with 95% confidence intervals (RT-PCR and antigen tests) of asymptomatic staff, patients/residents, and visitors in healthcare facilities in relation to 100,000 inhabitants for all seven administrative districts in the German state of Bavaria.

The five Bavarian districts with foreign borders to Czech Republic, Austria, and Switzerland (in total 1200 km) reported more tests per 100,000 inhabitants than those with inner German borders only (Middle and Lower Franconia). As evident in Figure 2A, Middle Franconia reported by far the fewest number of tests for asymptomatic persons in healthcare institutions relative to the size of the population as compared to the remaining districts. In contrast, and probably due to the lower number of tests, the antigen and RT-PCR positivity rates were observed to be the highest in Middle Franconia (Figure 2B).

### 3.4. Temporal Variation of Screening Test Results and Positive Rates

**Figure 3:**
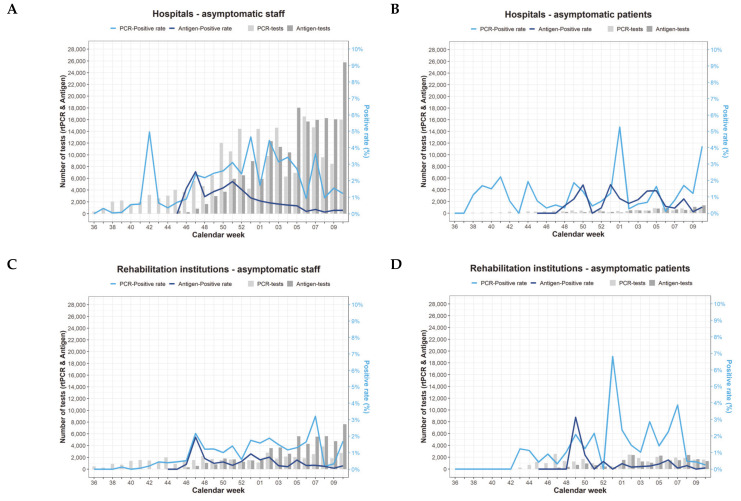

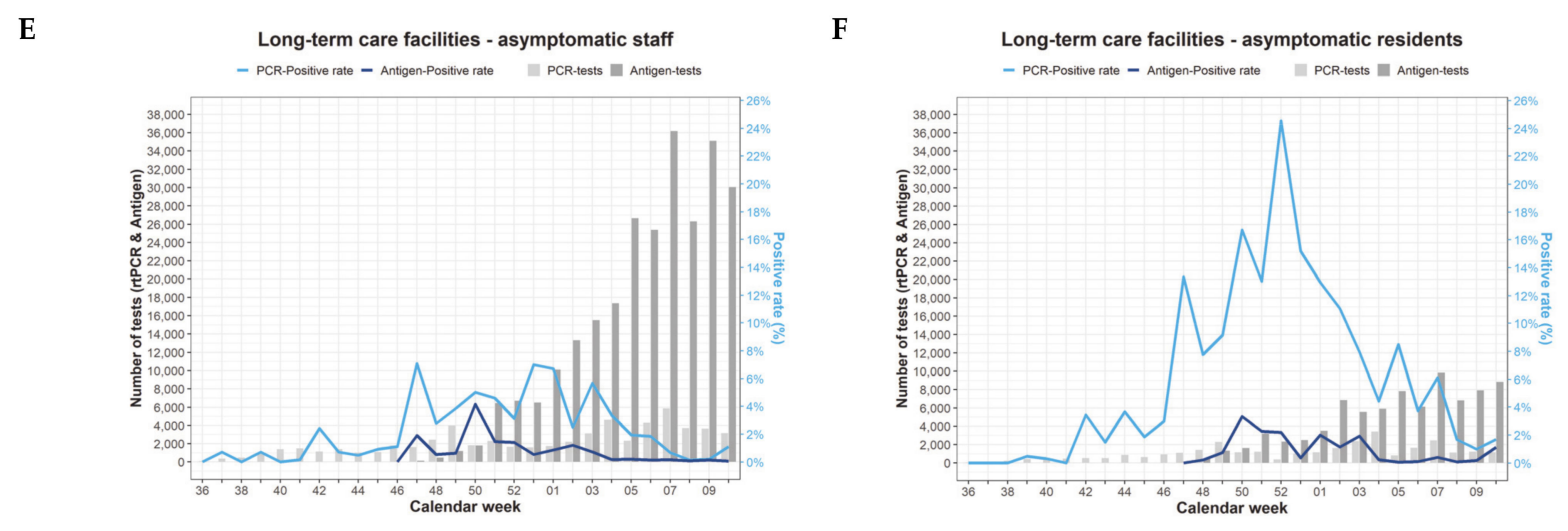
(**A**–**F**) Temporal patterns of the weekly number of real-time polymerase chain reaction (RT-PCR; light grey bars) and antigen tests (dark grey bars) and the proportion of positive RT-PCR (dark blue line) and antigen tests (light blue line) among asymptomatic staff (left diagrams) and patients/residents (right diagrams) in hospitals (**A**,**B**), rehabilitation institutions (**C**,**D)** and long-term care facilities (**E**,**F**).

**Figure 4:**
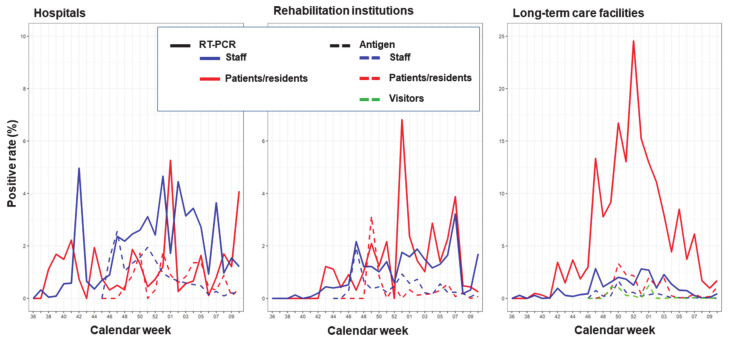
From September 2020 to March 2021, the weekly percentage of positive SARS-CoV-2 RT-PCR tests (solid lines) and positive SARS-CoV-2 antigen tests (broken lines) taken in asymptomatic staff, patients/residents, and visitors are presented, stratified by three types of healthcare institutions in the state of Bavaria, Germany. For visitors, only the antigen positivity tests in care facilities are shown due to an insufficient number of tests available for visitors of rehabilitation institutions and hospitals.

According to Figure 3 with respect to hospitals and rehabilitation institutions, a considerable increase in the reported antigen testing was observed for asymptomatic staff of hospitals (Figure 3A). There was further an elevated reporting of RT-PCR testing among asymptomatic staff observed for hospitals (Figure 3A) but not for rehabilitation institutions (Figure 3C). Since November 2020, the RT-PCR positivity rates have been consistently higher than the antigen positivity rates in asymptomatic staff (Figure 3A,C), except for asymptomatic patients in hospitals (Figure 3B and Figure 4), as compared to patients in rehabilitation institutions (Figure 3D and Figure 4).

The highest amount of reported antigen testing among all three types of institutions was observed for asymptomatic staff in LTC facilities (Figure 3E). Further, the RT-PCR positivity rate among asymptomatic residents of LTC facilities (Figure 3F and Figure 4) was considerable higher as compared to staff of LTC facilities (Figure 3E and Figure 4), also in comparison to patients in hospitals and rehabilitation institutions (Figure 3B,D). In contrast, no major variation was found in the antigen positivity rates across all types of healthcare institutions (Figure 3 and Figure 4).

**Figure 5:**
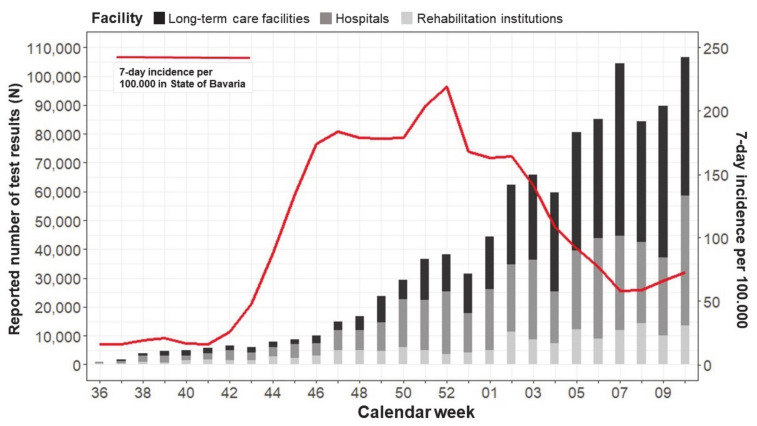
From September 2020 to March 2021, the combined weekly number of reported PCR and antigen tests for asymptomatic staff, patients/residents, and visitors of healthcare institutions are presented (bars). These tests were conducted as part of the voluntary SARS-CoV-2 screening program for hospitals, rehabilitation institutions, and care facilities for seniors and handicapped persons of the state of Bavaria, Germany. For the same period, the 7-day incidence per 100,000 inhabitants in Bavaria is presented (red line).

When aligning the test results with the official 7-day incidence per 100,000 inhabitants of the state of Bavaria, a shift to increasing RT-PCR and antigen testing was noticed after the incidence peak was reached in calendar week 52 around Christmas. This was most evident for hospitals and LTC facilities, most probably as a consequence of the enacted infections prevention measures (Figure 5).

## 4. Discussion

### 4.1. Summary of the Main Findings

Our evaluation of the online database included the first million screening test results of asymptomatic persons in healthcare institutions, covering all seven districts and 89% of all counties/cities in the state of Bavaria. In general, SARS-CoV-2 RT-PCR and antigen testing was most prevalent for the asymptomatic healthcare workforce as compared to patients, residents, or visitors in hospitals and LTC facilities. We observed the highest RT-PCR positivity rate among asymptomatic persons in LTC facilities, most pronounced for asymptomatic residents. Overall, the RT-PCR positivity were often higher than the antigen positivity rates in asymptomatic staff and patients/residents of all types of institutions. A major increase in the reported antigen testing was most evident for asymptomatic staff and residents in LTC facilities, reflecting the enacted policy measures.

### 4.2. Findings in Comparison to Previous Studies

In the present investigation, we evaluated SARS-CoV-2 testing of asymptomatic persons who are considered under increased risk for a SARS-CoV-2 infection and/or possibly a higher morbidity and mortality due to a more severe disease course of COVID-19 [7,9]. Between 40% and 45% of SARS-CoV-2 infections can be attributed to persons who were asymptomatic at the time of testing [32], emphasizing the risk of undetected silent transmission [8]. In fact, in our study, we observed a considerably high RT-PCR positivity rate at about 10%–20% during December 2020 and early January 2021 among asymptomatic residents in LTC facilities. This is in line with other findings regarding SARS-CoV-2 RT-PCR testing of asymptomatic residents in LTC facilities [10]. For instance, COVID-19 outbreak monitoring in a French nursing home during the first wave of the pandemic showed that one quarter of the residents who tested positive for SARS-CoV-2 were asymptomatic or had only mild symptoms [33]. Similarly, a study in the central German city of Frankfurt (Main) revealed that 41% of the positive nursing home residents and 12% of the positive staff were asymptomatic [34]. A study including 303 asymptomatic residents and healthcare workers from four LTC facilities in San Francisco (USA) showed that 40% of the asymptomatic residents tested positive for SARS-CoV-2 [13]. In the same study, 16% of the asymptomatic healthcare workers tested positive, emphasizing also their increased risk of infection as well as a possible source of transmission. In our study, the cumulative RT-PCR positivity rate for asymptomatic staff was 2.2% in hospitals and 1.0% in rehabilitation institutions. This corresponds to investigations from the UK looking at suspected asymptomatic carriage of SARS-CoV-2 among healthcare workers, with the asymptomatic RT-PCR positivity rates ranging between 0.3% and 3% [6,35].

### 4.3. Asymptomatic Testing as a Surveillance Instrument

Our findings underpin the assumption that asymptomatic infections might add notably to the overall SARS-CoV-2 transmissions, in particular among vulnerable population groups in LTC facilities where social distancing is aggravated. Asymptomatic transmission also has been indicated as the “Achilles heel” [36] of the current strategies. This is supported by a current systematic review suggesting that at least one third of confirmed SARS-CoV-2 infections are asymptomatic [37]. Therefore, universal mass-testing strategies as targeted infection prevention concepts might represent an effective measure preventing onward transmission of SARS-CoV-2 to residents, patients, and the healthcare workforce [32,38].

The increasing incidence in the federal state of Bavaria in autumn 2020 led the State Ministry of Health and Care issue several infection-protection measures to control the rising infection rates and to prevent further spread of COVID-19 in the community. In November 2020, the district administrative authorities were able to implement voluntary mass screenings for a range of facilities when the 7-day incidence exceeds 200 per 100,000 inhabitants [39]. This particularly concerned LTC facilities and hospitals. Since the 8th of December 2020 [40,41], it became obligatory for employers running LTC facilities and ambulatory/mobile nursing services to perform regular testing at least twice a week among their healthcare staff. In addition, visitors to LTC facilities had to present a negative RT-PCR or antigen SARS-CoV-2 test result, fulfilling the test requirements of the national public health (Robert Koch) institute, in addition to the commonly enacted hygiene measures, including wearing FFP2 (Filtering Face Piece 2) masks. The implication of the enacted measures is reflected in our data, with the coincident increase in reported RT-PCR and antigen testing, in particular for hospitals and LTC facilities. Since a peak at the end of 2020, we noted a decline in residents being tested positive for SARS-CoV-2. We can only speculate whether the lagged decline was associated with the enforced surveillance testing among asymptomatic healthcare workforce as the suggested main source of infection transmission or whether this was related to the simultaneous establishment of strict quarantine measures and the start of extensive COVID-19 vaccinations in LTC facilities, which were highly prioritized [42]. Unfortunately, the contribution of each measure in relation to the declining positive rates cannot be estimated and, therefore, has to be interpreted with caution.

### 4.4. Comparison of RT-PCR and Antigen Positivity Rates among Asymptomatic Populations

The RT-PCR screening tests detected infections with SARS-CoV-2 in 1.0–2.2% of asymptomatic healthcare professionals and patients of hospitals and rehabilitation institutions, and in 7.1% of asymptomatic residents of LTC facilities. Reasons for the differences in the magnitude of the RT-PCR positive rates might be that some of the tested asymptomatic residents were actually pre-symptomatic and/or limited in communicating possible symptoms due to the severity of their health condition, or that the workforce was less aware of symptoms due to increased workload [15,36,43]. It is also suspected that a considerable number of persons who tested positive by the antigen test were re-tested positive by RT-PCR but that their results were entered into a database not necessarily of the same facility. Second, it has been observed that extensive testing is positively related to a decline in infections and therefore a good measure to reduce the proportion of positives before an outbreak situation escalates [44,45]. Indeed, the amount of testing of staff (both by RT-PCR and antigen tests) was higher than that for residents; the percentage of positives, however, was lower in staff than in the residents. Identifying positive but asymptomatic staff therefore helped preventing outbreaks among the healthcare workforce and consequently in residents.

In light of the enacted national test strategy in October 2020 [24] and the infection control measures in the State of Bavaria [41], the application of antigen tests is now being recommended for mass testing and widely applied among asymptomatic healthcare workers and visitors in LTC facilities. In Germany, only antigen tests with a diagnostic sensitivity (i.e., true positive rate) of over 80% are officially licensed and eligible for reimbursement through the Coronavirus-Testverordnung (TestV) [46]. Although favourable in implementation and time to result, there remain issues with respect to specificity and particularly sensitivity in comparison to RT-PCR tests, the diagnostic gold standard [47,48].

The RT-PCR positivity rates in staff and residents were more variable than the antigen positivity rates, which apart from being generally lower by one order of magnitude also showed a lower variance. This reflects the higher sensitivity of the RT-PCR assay, able to pick up low viral RNA levels in the pre-infectious and post-infectious phases of asymptomatic infections, whereas antigen tests have been shown to score positive in the infectious phase with high viral RNA levels only [49,50]. Proper adherence to hygiene measures is therefore imperative to compensate for the lower diagnostic sensitivity of antigen tests, in particular among asymptomatic populations.

In this context, population-wide antigen mass-screening, as performed in Slovakia, or among specific asymptomatic population groups, might have the potential to identify a considerable number of potential infectious individuals with no or mild symptoms at a larger scale, in turn preventing further short-term transmission [51,52,53,54]. However, it remains unclear how often mass-screening efforts of a whole population has to be repeated to achieve sustainable effects in the containment of the spread of SARS-CoV-2. As mentioned above, evidence regarding the clinical performance of rapid antigen tests in asymptomatic individuals is still scarce and, therefore, available results should be interpreted with caution [48]. Nevertheless, 2327 positive antigen tests of asymptomatic individuals were entered in our online database. Considering that more asymptomatic persons tested positive, but which were not reported to us, we speculate that a number of infection outbreaks in Bavarian healthcare institutions may have been prevented based on the relatively inexpensive and fast antigen tests.

### 4.5. Strengths and Limitations

The key strengths of our study are the size of the online database, including a considerable amount of RT-PCR and antigen test results of asymptomatic persons from healthcare institutions, the long assessment period of more than seven months, and the regional spread across the large state of Bavaria. To our knowledge, there is no comparable database established aiming at monitoring infection patterns comprehensively in pre- or asymptomatic person groups by voluntary and free tests in healthcare institutions. The Bavarian online database further monitors a range of different types of facilities critical in relation to the pandemic, encompassing vulnerable individuals.

One of the main limitations of our study is that the database does not allow head-to-head comparisons of antigen and RT-PCR test results, thus evaluating the diagnostic accuracy of the antigen tests in comparison to the gold standard RT-PCR. To ensure high compliance by the healthcare institutions and avoid potential data protection regulation issues, we decided to collect only anonymous data of generally healthy persons and without further socio-demographic information, e.g., sex and age. Nevertheless, according to our data, some potentially asymptomatic viral transmission must have been prevented to a certain extent by the use of antigen tests in asymptomatic individuals, thus justifying this testing strategy. A further limitation of the online database is that not all healthcare institutions in Bavaria have (regularly) reported their RT-PCR and antigen test results from screening their asymptomatic staff, patients, residents, and visitors.

In this context, the online database might therefore represent a more general monitoring tool rather than yield insights at the facility level. The purpose of the online database was primarily to monitor the efficiency of the implementation of the testing directive. Daily instead of weekly reporting, however, would be more helpful to monitor the implementation. Although data entry is compulsory for the institutions, non-compliance has not been sanctioned and a number of facilities have not been reporting or did so only with some delay. Moreover, the initial focus of the online database was to acquire the testing data of the healthcare workforce. Test results from patients, residents, and visitors within the selected facilities were only recorded under certain conditions, such as patient admission, and are therefore not representative of the total patient population. However, recent reminders by the State Ministry of Health and Care distributed via the local public health offices to all healthcare institutions have led to further increases in the number of institutions now reporting their screening results. Thus, future evaluations of the screening test data will be based on an even higher coverage of institutions across the state and will allow more representative results of subgroups, such as visitors, who may also contribute to viral spread in healthcare institutions.

## 5. Conclusions

Our results indicated that in LTC facilities and hospitals with extremely vulnerable patients and residents, infection surveillance of asymptomatic persons based on RT-PCR tests and antigen testing may facilitate the long-term management of COVID-19. In fact, during the second wave of the current pandemic, we found the highest RT-PCR positivity rates among asymptomatic residents as compared to asymptomatic staff or visitors. Reflecting the enacted policy measures, the increase in reported antigen testing was strongest for asymptomatic staff and residents of LTC facilities as compared to other healthcare institutions. It will be a continuous challenge for a screening database like ours to collect sufficient data from various public and private healthcare institutions by keeping the motivation of the users on a high level. This is especially relevant where institutions get reimbursed only for the testing material but not for entering the testing data, and also in times where incidences and the general interest in efforts to fight the pandemic are low. Regular feedback to not only the authorities but also to individual institutions may be one approach to ensure wide coverage, thus providing representative data of the mass testing during a pandemic for both scientific use and policy decision.

## Figures and Tables

**Table 1:** Overview of accumulated SARS-CoV-2 screening tests and positive rates (grey columns) among asymptomatic persons in healthcare institutions in Bavaria, Germany.

	RT-PCR-Tests ^#^ N (%)	Positive n	Positive (%) (95%CI)	Antigen Tests ** N (%)	Positive n	Positive (%) (95%CI)
OVERALL (100%) *	382,240 (37%)	8380	2.2% (2.1–2.2)	655,906 (63%)	2327	0.4% (0.3–0.4)
Hospitals	213,170 (100%)	4673	2.2% (2.1–2.3)	196,723 (100%)	1013	0.5% (0.5–0.5)
Staff	201,826 (95%)	4,511	2.2% (2.2–2.3)	180,350 (92%)	942	0.5% (0.5–0.6)
Patients	10,940 (5%)	147	1.3% (1.1–1.6)	8,288 (4%)	52	0.6% (0.5–0.8)
Visitors	404 (0.2%)	15	3.7% (2.3–6.1)	8,085 (4%)	19	0.2% (0.2–0.4)
**Rehabilitation institutions**	76,244 (100%)	923	1.2% (1.1–1.3)	77,166 (100%)	256	0.3% (0.3–0.4)
Staff	46,779 (61%)	508	1.1% (1.0–1.2)	53,981 (70%)	184	0.3% (0.3–0.4)
Patients	29,029 (38%)	412	1.4% (1.3–1.6)	20,724 (27%)	66	0.3% (0.3–0.4)
Visitors	436 (0.6%)	3	0.7% (0.2–2.1)	2,461 (3%)	6	0.2% (0.1–0.5)
**Long-term care facilities**	92,826 (100%)	2784	3.0% (2.9–3.1)	382,017 (100%)	1058	0.3% (0.3–0.3)
Staff	61,450 (66%)	602	1.0% (0.9–1.1)	259,534 (68%)	377	0.1% (0.1–0.2)
Residents	30,758 (33%)	2181	7.1% (6.8–7.4)	80,808 (21%)	636	0.8% (0.7–0.9)
Visitors	618 (0.7%)	1	0.2% (0.02–1.1)	41,675 (11%)	45	0.1% (0.08–0.1)

**^#^** Since the last week of July 2020; * due to rounding, the percentages do not always sum up to 100%, ** since the first week of November 2020 (calendar week 45); 95%CI = 95% confidence interval.

## Data Availability

The data presented in this study are only available on request from the corresponding author. In general, the data are not publicly available due to confidentiality concerns and cannot be provided without agreement of the Bavarian State Ministry of Health and Care (Germany).
